# A Trend Forecasting Method for the Vibration Signals of Aircraft Engines Combining Enhanced Slice-Level Adaptive Normalization Using Long Short-Term Memory Under Multi-Operating Conditions

**DOI:** 10.3390/s25072066

**Published:** 2025-03-26

**Authors:** Jiantao Lu, Kuangzhi Yang, Peng Zhang, Wei Wu, Shunming Li

**Affiliations:** 1 College of Energy and Power Engineering, Nanjing University of Aeronautics and Astronautics, Nanjing 210001, China; yangkz@nuaa.edu.cn (K.Y.); wu_wei@nuaa.edu.cn (W.W.); 2 AECC Guiyang Engine Design Research Institute, Guiyang 550081, China; kevin_diab@163.com; 3 School of Automotive Engineering, Nantong Institute of Technology, Nantong 226002, China; smli@nuaa.edu.cn

**Keywords:** trend forecasting, *L*_1_ filtering, enhanced SAN, LSTM, multi-operating condition

## Abstract

**Highlights:**

Addressing the challenges in the forecasting tasks of non-stationary signals in aircraft engines, a forecasting method using enhanced SAN and LSTM under multi-operating conditions is proposed in this paper. This study adopts a condition recognition technology to identify the operating conditions. *L*_1_ filtering is used to extract the trend part and give a quantitative estimation of slice length in enhanced SAN. Furthermore, a tail amendment technology is added to expand the application of enhanced SAN. The validity of the proposed method is verified by the test data of an aircraft engine, and the results show the effectiveness and superiority of the proposed method.

**What are the main findings?**

- Multi-operating conditions are recognized.
- Slice length is estimated.
- Tail amendment technology is adopted.

**What are the implications of the main findings?**

- Condition recognition technology identifies the operating conditions and divides the conditions into several models for training and testing.
- Compared with the choices of slice length based on experience in traditional SAN, *L*_1_ filtering gives a quantitative estimation of slice length by the second-order difference, offering more specificity towards analyzed data.
- Tail amendment technology expands the application range of enhanced SAN, allowing it to deal with data of different sequence lengths.

**Abstract:**

Trend forecasting and early anomaly warnings are important for avoiding aircraft engine failures or accidents. This study proposes a trend forecasting method based on enhanced Slice-level Adaptive Normalization (SAN) using a Long Short-Term Memory (LSTM) neural network under multi-operating conditions. Firstly, a condition recognition technology is constructed to automatically identify the operating conditions based on the predetermined judgment conditions, and vibration signal features are adaptively divided into three typical operating conditions, namely, the idling operating condition, the starting operating condition and the utmost operating condition. The features of original signals are extracted to reduce the impacts of signal fluctuations and noise preliminarily. Secondly, enhanced SAN is used to normalize and denormalize the features to alleviate non-stationary factors. To improve prediction accuracy, an *L*_1_ filter is adopted to extract the trend term of the features, which can effectively reduce the overfitting of SAN to local information. Moreover, the slice length is quantitatively estimated by the fixed points in *L*_1_ filtering, and a tail amendment technology is added to expand the applicable range of enhanced SAN. Finally, an LSTM-based forecasting model is constructed to forecast the normalized data from enhanced SAN, serving as input during denormalization. The final results under different operating conditions are the output from denormalization. The validity of the proposed method is verified using the test data of an aircraft engine. The results show that the proposed method can achieve higher forecasting accuracy compared to other methods.

## 1. Introduction

Aircraft engines play an important role in the aviation industry, and their health status closely relates to the safety and stability of the aircraft. However, aircraft engines are prone to abnormality and failure under varying operating conditions, which may lead to significant economic losses and even casualties. The vibration signals of aircraft engines contain abundant abnormal information. Therefore, vibration signals are critical for vibrational characteristic analysis, which can be used for fault diagnosis [1]. Some indicators of vibration signals, such as vibration features, can reveal the health state of aircraft engines [2]. Generally, when an aircraft engine works in normal conditions, these indicators are relatively stable with small fluctuations. When an aircraft engine shows abnormality and failures, these indicators will undergo significant changes, even sharp increases or decreases. Therefore, we can develop a forecasting method to analyze the trends in vibration signal features and identify early abnormalities, which can provide an early warning of possible machinery faults by referencing the forecasting part, and is significant for ensuring the safety and reliability of aircraft engines [3].

Forecasting methods can be used to estimate future data with historical information. Trend-forecasting methods can be divided into three main types: model-based forecasting methods, knowledge-based forecasting methods and data-based forecasting methods [4]. One precondition of model-based forecasting methods is to obtain an accurate mathematical model and fault model of vibration components. The main advantage of model-based forecasting methods is that they can meet real-time requirements. However, aircraft engine signals are usually found in complex nonlinear vibration systems, and vibration signals and vibration faults are affected by many factors. Therefore, it is time-consuming and laborious to build a suitable and accurate mathematical model [5], which means that model-based forecasting methods cannot be used easily in practical applications. Knowledge-based forecasting methods do not need an accurate mathematical model, making full use of expert knowledge and experience in various subjects. Two typically applied types of knowledge-based forecasting are expert system forecasting and fuzzy logic forecasting [6]. One disadvantage of expert system forecasting is that knowledge bases have limited fault patterns. If no matching rule exists with symptoms in the knowledge base, this can easily result in misdiagnosis or the failure of diagnosis. Fuzzy logic forecasting can use expert knowledge to construct a fuzzy rule base, and can make full use of expert knowledge and experience. However, it is difficult to choose a suitable membership function for fuzzy logic [7]. Data-based forecasting is mainly based on machine learning. A forecasting model can be trained using collected historical monitoring data, and future results can be forecasted by the trained model. Traditional machine learning methods include Support Vector Machine (SVM) [8], Gradient Boosting Regression Tree (GBRT) [9], Extreme Gradient Boosting (XGBoost) [10], Autoregressive Integrated Moving Average (ARIMA) [11], and so on. These methods were applied to data-based forecast methods early on. With the development of deep learning, many deep learning methods have been used to construct forecasting models. Deep neural networks have been widely applied in time series forecasting [12]. Artificial Neural Networks (ANNs) play a significant role in time series prediction due to their universal approximation ability, nonlinear modeling characteristics, and use of data-driven training models [13,14]. A backpropagation (BP) neural network is applied for forecasting as well [15]. The Recurrent Neural Network (RNN) is a typical neural network used in time series forecasting, which can be utilized to capture the temporal dependence by summarizing the past information in the time series [16]. However, some scholars found that RNN suffers from a problem of gradient explosion [17]. The Long Short-Term Memory (LSTM) neural network is utilized to address that problem in RNN [18]. The neural network algorithms used in deep learning, such as Convolutional Neural Network (CNN) [19], the LSTM neural network, and so on, have achieved good results in forecasting applications [20]. Other algorithms like Gated Recurrent Unit (GRU) [21] and the Bidirectional Long Short-Term Memory Network (Bi-LSTM) [22] are developed on the basis of LSTM. Moreover, the appearance of attention has been used to develop a widely used model—Transformer [23]. Many variants have also spawned from this. Informer [24] improves upon the attention mechanism of Transformer to process long sequences more efficiently. Autoformer [25] introduces a decomposition mechanism based on Transformer. DLinear [26] is introduced as a simpler model compared with Transformer. This data-driven prediction method does not rely on establishing accurate prior models, and can be flexibly applied based on historical data, achieving higher prediction accuracy and greater feasibility in handling prediction problems.

However, directly processing the original signal during forecasting will cause the forecasting model to overfit local information to some extent [27]. Generally, the vibration signals collected from the aircraft engine may contain non-stationary factors. Therefore, the difference of distribution between input and horizon series, which is called distribution shift, will reduce the forecasting accuracy. To address the non-stationary forecasting task, a forecasting model with special normalization is needed. Spatial and Temporal Normalization (NST) [28] is one of the special normalization methods that extracts high-frequency components and local components from the original data through both temporal and spatial modules, respectively. However, the distribution of the sequence is considered to be constant in NST. Reversible Instance Normalization (RevIN) [29] is another normalization method that adopts two layers to decompose and forecast the result. Similarly, RevIN has the same drawbacks as NST. The distribution shift in Time Series (Dish-TS) [30] tries to learn the distribution in the future, but the complicated learning stage adversely affects the forecasting result. Among those normalization methods, Slice-level Adaptive Normalization (SAN) [31] uses two-stage training schema, while RevIN and NST use one-stage training schema. The two-stage training schema in SAN independently trains the evolving trend from the statistic property and deals with the forecasting task by divide and conquer, which makes it more robust and suitable for non-stationary tasks. On the other hand, the data at the slice level allows for the better capturing of local distribution in the future. The slice length of SAN is a very important adjustable parameter that affects the forecasting result. Traditional SAN adopts experience values for slice length and lacks the specificity of current data, and this may lead to a poor prediction.

To address these problems, this paper proposes a forecast method based on enhanced SAN using the LSTM neural network, which can be used to predict the trend of aircraft engine performance under different operating conditions. Firstly, a condition recognition technology is used to identify the typical operating conditions of vibration signal features. Secondly, enhanced SAN is used to normalize and denormalize the features to alleviate non-stationary factors. *L*_1_ filtering [32] is adopted to extract the trend of the features to capture global information. Moreover, the slice length is quantitatively estimated by the fixed points in *L*_1_ filtering, and a tail amendment technology is added to expand the applicable range of enhanced SAN. Finally, an LSTM-based forecasting model is constructed to forecast the normalized data from enhanced SAN, and the data from the LSTM model will be used as the input of denormalization. The final results under different operating conditions are the output from denormalization. The validity of the proposed method is verified by the test data for an aircraft engine. The results show that the proposed method can achieve higher forecasting accuracy than comparative methods in almost all error indicators.

The main contributions of the proposed method are as follows:

1. A condition recognition technology is adopted for identifying three typical operating conditions automatically;
2. The trend parts of the signals are extracted with *L*_1_ filtering in enhanced SAN and the slice length is quantitatively estimated by the second-order difference of the fixed points in *L*_1_ filtering;
3. A tail amendment technology is used in the last incomplete slice of enhanced SAN, extending the application range of the method.

The rest of this paper is organized as follows. Section 2 presents the basic theory of *L*_1_ filtering, SAN and LSTM. Section 3 describes the proposed method in detail. Section 4 gives the validation of the proposed method with an aircraft engine dataset. Section 5 summarizes the conclusions.

## 2. Basic Theory

### 2.1. L_1_ Filtering

*L*_1_ filtering is a variation on Hodrick–Prescott (H-P) filtering. The characteristic of *L*_1_ filtering is that the trend estimates of filtering are smooth in the sense of being piecewise linear, making this an effective method for estimating the trend components that are piecewise linear from time series.

Suppose here that [X(1), X(2),⋯,X(t)] includes the trend part [Y(1), Y(2),⋯,Y(t)] and the residual part [Z(1), Z(2),⋯,Z(t)], that is, Z(t)=X(t)−Y(t). *L*_1_ filtering is used to optimize the trend part and the residual part. The ultimate goal of *L*_1_ filtering is that the trend part needs to be smooth and the residual part should be as small as possible. The objective function of *L*_1_ filtering can be formulated as:

(1) $$\min\;\;\sum_{n=1}^{t}{\left[X(n)-Y(n)\right]}^{2}+\lambda \sum_{n=2}^{t-1}\left|Y(n-1)-2Y(n)+Y(n+1)\right|$$

where *X*(*n*) is the feature extracted from the original vibration signal, *Y*(*n*) is the trend part of the feature and λ is an adjusting parameter. The useful information is often contained in *Y*(*n*). Meanwhile, the LSTM neural network can perform better with filtered features.

The filtering effect can be determined by adjusting parameter λ. The larger λ is, the more smoothly the trend part performs, which means that the filtering can concentrate more on global information, but ignores the detail. Conversely, a small λ will cause filtering concentrate on local information, but neglect the trend.

Considering the residual part [Z(1), Z(2),⋯,Z(t)], the optimization objective function can be written in matrix form as

(2) $$\min\;\;{\left\|Z\right\|}_{2}^{2}+\lambda {\left\|DY\right\|}_{1}$$

where D is the Toeplitz matrix, ${\left\|DY\right\|}_{1}$ denotes the *L*_1_ norm of DY, and ${\left\|Z\right\|}_{2}$ denotes the *L*_2_ norm of *Z*. λ is an adjustable parameter, and it controls the proportion of global part and local part.

The signal filtered by *L*_1_ filtering will exhibit a piecewise linear feature. The fixed points between different linear parts are dividing points of their respective segments, where the signal in each segment displays a linear feature.

### 2.2. Slice-Level Adaptive Normalization

SAN is primarily proposed to address the non-stationary data distribution issue. Considering the distribution difference between the input sequence and the horizon sequence, directly normalizing the output part using the input part may cause a prediction bias. SAN uses slicing normalization to remove non-stationary factors and assists the forecasting models in predicting better results with input series. To estimate the evolving distribution for each horizon slice, SAN applies a two-layer perceptron network to learn statistics distribution for a future slice.

We assume that the input series x are split into *R* non-overlapping slices ${\left\{x_{j}\right\}}_{j=1}^{R}$ equally, and the horizon series split with the same slice length as well. The statistics prediction [31] procedure with Multi-Layer Perceptron (MLP) can be described as

(3) $$\hat{\mu }=W_{1}*f_{\mathrm{MLP}}(\mu -\rho ,\bar{x}-\rho )+W_{2}*\rho ,\;\;\hat{\sigma }=f_{\mathrm{MLP}}(\sigma ,\bar{x})$$

where $\mu =\{\mu_{1},\mu_{2}\cdots \mu_{R}\}$ and $\sigma =\{\sigma_{1},\sigma_{2}\cdots \sigma_{R}\}$ are the mean and standard deviation of *R* input slices, respectively. ρ denotes the overall mean of input series, while $\hat{\mu }$ and $\hat{\sigma }$ represent the mean and standard deviation prediction of output slices, respectively. $\bar{x}$ is the normalized series. $W_{1}$ and $W_{2}$ are learnable vectors. $f_{\mathrm{MLP}}$ is MLP layers.

The standard deviation is predicted via a similar method. The error between the statistics prediction parameter and the real statistic parameter is used as the loss function of the backpropagation network. Since the normalization flow of SAN serves in the capacity of a constraint for the forecasting model, the integrity forecast process is a bi-level optimization problem. The overall training procedure can be formulated as

(4) $$\begin{array}{l} \arg\;\underset{\theta }{\min}\sum_{\left(x,y\right)}l_{fm}(\theta ,\phi^{*},(x,y)),\\ \;\;\;s.t.\phi^{*}=\arg\;\underset{\phi }{\min}\sum_{\left(x,y\right)}l_{sp}(\theta ,\phi^{*},(x,y)). \end{array}$$

where $l_{fm}$ represents the loss of the forecasting model, $l_{sp}$ represents the loss of statistics prediction, ϕ and θ are weight parameters in the corresponding training procedure, respectively. The primary objective at the upper level is to achieve accuracy in forecasting results with respect to ground truth, and the goal at the lower level is to ensure the similarity in distribution between the normalized output and ground truth.

The training paradigm of the forecasting model with SAN can be decoupled into two stages. Firstly, an estimation of the horizon distribution can be trained by using stochastic gradient descent. Secondly, the forecasting model trains while the statistics prediction model is frozen. The forecasting model can handle the simpler task with normalized data after statistics prediction.

### 2.3. Long Short-Term Memory Neural Network

**Figure 1:**
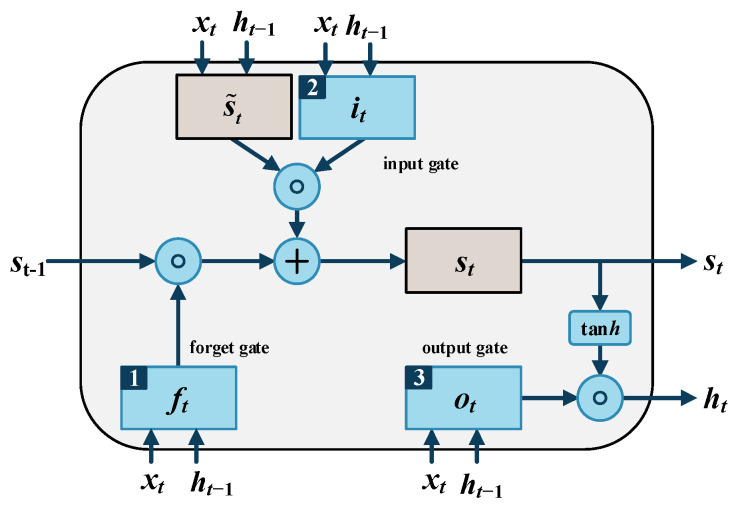
Structure of LSTM memory cell.

The LSTM neural network evolved from the Recurrent Neural Network (RNN). It consists of one input layer, one output layer, and one or more hidden layers. A hidden layer of the LSTM neural network contains many neurons called memory cells. Each memory cell includes three gates: an input gate, an output gate and a forget gate, denoted by $i_{t}$, $o_{t}$ and $f_{t}$, respectively. The structure of the memory cell is shown in Figure 1.

At every *t* moment, each gate receives and handles the input $x_{t}$ now and the output $h_{t-1}$ from the last moment. The three kinds of gates have different purposes, numbered 1, 2 and 3 in Figure 1. Forget gate decides which information to be deleted and which to commit to memory. The input gate controls the input information. The output gate controls the information delivered to the next memory cell.

Suppose $x_{t}$ is the input vector at moment *t*, $h_{t}$ is the output vector at moment *t*, $W_{f,x}$, $W_{f,h}$, $W_{\tilde{s},x}$, $W_{\tilde{s},h}$, $W_{i,x}$, $W_{i,h}$, $W_{o,x}$ and $W_{o,h}$ are weight matrixes, $b_{f}$, $b_{\tilde{s}}$, $b_{i}$ and $b_{o}$ are bias vectors, $f_{t}$, $i_{t}$ and $o_{t}$ are activation vectors, and $s_{t}$ and ${\tilde{s}}_{t}$ represent the state of the cell and the state of the candidate cell, respectively. The calculation of parameters at moment t proceeds as follows.

In the forget gate, the unit decides which part of information is useless based on the current input, the output of the *t* − 1 time storage unit, and the bias of the forget gate. It calculates the activation vector $f_{t}$ of the gate at moment *t* from the input $x_{t}$, the output $h_{t-1}$ at last moment, and the bias $b_{f}$. Then, it scales the activation vector to a range from 0 to 1 via a sigmoid activation function. The calculation procedure can be formulated as

(5) $$f_{t}=sigmoid\left(W_{f,x}x_{t}+W_{f,h}h_{t-1}+b_{f}\;\right)$$

In the input gate, the unit decides which part of the information needs to be added to the state of the cell. Initially, it calculates the state of the candidate cell as

(6) $${\tilde{s}}_{t}=\tanh\left(W_{\tilde{s},x}x_{t}+W_{\tilde{s},h}h_{t-1}+b_{\tilde{s}}\right)$$

Then, it calculates the activation vector $i_{t}$ as

(7) $$i_{t}=sigmoid\left(W_{i,x}x_{t}+W_{i,h}h_{t-1}+b_{i}\right)$$

After finishing the calculation of the forget gate and input gate, the state of the cell updates as

(8) $$s_{t}=f_{t}\circ s_{t-1}+i_{t}\circ {\tilde{s}}_{t}$$

where ∘ is the Hadamard product.

In the output gate, the output vector $h_{t}$ is calculated via the following formulae

(9) $$o_{t}=sigmoid\left(W_{o,x}x_{t}+W_{o,h}h_{t-1}+b_{o}\right)$$

(10) $$h_{t}=o_{t}\circ \tanh\left(s_{t}\right)$$

Finally, LSTM updates its weight parameters using these memory cells.

## 3. Proposed Method Based on Enhanced SAN Using LSTM

The actual aircraft engine vibration signals are often non-stationary and contain random fluctuations, which will reduce the subsequent prediction accuracy. To mitigate the above effects, this paper proposes a trend forecast method-based enhanced SAN using the LSTM neural network under multi-operating conditions. The proposed method mainly contains two sections named condition recognition and enhanced SAN, as follows.

### 3.1. Condition Recognition

Aircraft engines usually run under varying operating conditions, which makes vibrational signals vary greatly in different distributions. Considering the changes in the operating conditions in aircraft engine vibration signals, a condition judgement is adopted in the proposed method to recognize the operating conditions, enabling the forecasting model to produce the corresponding results under different operating conditions.

To represent the main operating conditions during the operation, three operating conditions are chosen to represent the typical scenario, namely, the idling operating condition, the starting operating condition and the utmost operating condition. The idling operating condition represents the idling state of engine, during which the rotation speed of the aircraft engine is relatively low. The starting operating condition represents the starting state of the engine, when the working mode of the engine changes from the community land mode to the air mode. The utmost operating condition represents the max throttle state of engine, that is, here, the power level angle controlled by the throttle is relatively high.

The discrimination indicators of the idling operating condition include the range of low-pressure rotor speed, the range of high-pressure rotor speed, and fluctuation values of low-pressure rotor speed and high-pressure rotor speed. The discrimination indicators of the starting operating condition include the state mark of the engine state between the entry mark and the exit mark. The discriminations of the utmost operating condition include the range of low-pressure rotor speed, the range of high-pressure rotor speed, and the fluctuation values of power level angle. In addition, the determination of the idling operating condition and the utmost operating condition needs to be maintained for a period of time. The recognition of the starting operating condition will be identified first, followed closely by the idling operating condition recognition, and subsequently by the utmost operating condition recognition.

A condition recognition technology is adopted to identify the operating conditions according to the above discrimination. Additionally, given that original vibrational signal contains noise with fluctuation, the features of the original signal are extracted for forecasting. The vibration total value in the front axle is extracted in the idling operating condition, the second harmonic component in the high-pressure rotor in the front axle is extracted in the starting operating condition, and the vibration component value in the front axle is extracted in the utmost operating condition. These are typical features of aircraft vibrational signals that can reflect the vibrational characteristics of the data. The condition recognition technology identifies the operation condition of the features through a certain length of data. After the recognition of the operating condition, the features of the aircraft engine in the operating condition will be input into the forecasting model under the corresponding operating condition, and will generate the forecasting result.

### 3.2. Enhanced Slice-Level Adaptive Normalization

To get rid of the effects of local information in traditional SAN, *L*_1_ filtering is used to extract the trend part of the original signal in enhanced SAN, which facilitates the calculation of the distribution parameters, with fewer details causing interference.

The actual data of vibration features may possess non-stationary factors, that is, the input series and horizon series may distribute in different levels. The effect of the additional SAN is to mitigate the weakness so that the distribution of the input and horizon parts in every sequence can perform to similar levels. The slice length of SAN is a key adjustment parameter that affects the forecasting result. However, traditional SAN adopts the slice length in experience values, which may lead to a poor prediction.

Moreover, considering that *L*_1_ filtering gives data in a linear-part form, the filtered data in each part seem to exhibit proximity in the distribution. The mean and standard deviation of the filtered data will be closer in each part compared with those calculated in a random section. Meanwhile, SAN performs statistics prediction with a fixed slice length, and calculates the estimation of mean and standard deviation in each slice. The mean provides the general scale of the slice, and the standard deviation shows the degree of dispersion. The slice length is an undetermined parameter in SAN, which has a considerable influence on statistics prediction.

**Figure 2:**
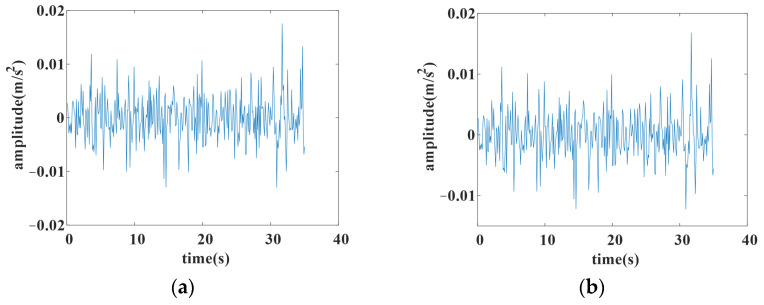

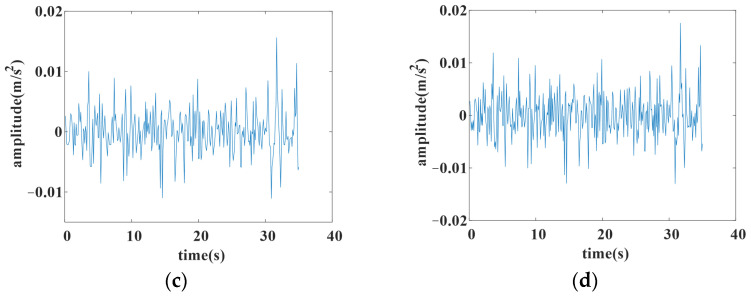
The result of *L*_1_ filtering with different lambda. (**a**) Original data; (**b**) filtered with lambda 0.0002; (**c**) filtered with lambda 0.0005; (**d**) filtered with lambda 0.001.

To better illustrate the effect of trend extraction by *L*_1_ filtering, an example of *L*_1_ filtering with different λ is given. The original data are shown in Figure 2a, and the filtered data with different λ are shown in Figure 2b, Figure 2c and Figure 2d, respectively.

Comparing the original data with the filtered ones, it can be argued that the larger λ is, the stronger the filtering effects, which suggests that the trend part is significantly concentrated. Meanwhile, more detailed information on the original data will be lost with a larger λ. Thus, λ should be assigned an appropriate value that can extract the trend part of the original data with less loss of local information. The choice of λ determines the expected data to predict in the forecasting model.

To give a quantitative estimation of slice length in SAN, a second-order difference *D*_2_ of the filtered aircraft engine vibration signal is calculated by *L*_1_ filtering,

(11) $$D_{2}=\Delta (\Delta z(x))$$

where Δ is a difference operation and *z*(*x*) is the signal filtered by *L*_1_ filtering.

**Figure 3:**
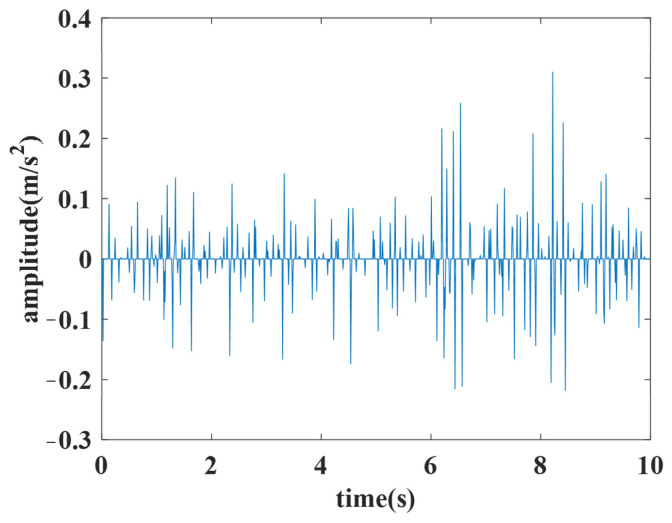
The second difference of filtered data.

The second-order difference *D*_2_ is used to find the fixed points, such as the dividing points between the linear parts in Figure 2b, in an obvious way. An example of *D*_2_ is shown in Figure 3. It can be easier to find the amount of dividing points that are almost at the peak of the part in the filtered data. Suppose the amount of fixed points of a filtered aircraft engine vibration feature is *M*, and the length of the original data is *L*; Q=L/M is regarded as an average interval length, adopted as a quantitative estimation of slice length.

Suppose that slice length is *Q*; enhanced SAN splits the original series ${\left\{x_{j}\right\}}_{j=1}^{R}$ into *R* slices, where R=L/Q. The mean and standard deviation for each slice can be calculated by

(12) $$\mu_{j}=\frac{1}{Q}\sum_{t=1}^{Q}x_{j,t},\;{\left(\sigma_{j}\right)}^{2}=\frac{1}{Q}\sum_{t=1}^{Q}{\left(x_{j,t}-\mu_{j}\right)}^{2}$$

where $x_{j,t}$ is the input vibration feature value of slice $x_{j}$ at moment *t*, and $\mu_{j}$ and $\sigma_{j}$ are the mean and standard deviation of slice $x_{j}$, respectively. Then, enhanced SAN normalizes the slices with the statistic parameters as

(13) $$\bar{x_{j}}=\frac{1}{\sigma_{j}+\varepsilon }\circ (x_{j}-\mu_{j})$$

where $\bar{x_{j}}$ is the normalized feature, ε is a small constant set to avoid the zero denominator, and ∘ is the Hadamard product. The normalized slices $\bar{x_{j}}$ are restored data without non-stationary factors that will be input into the forecasting model LSTM.

The statistics prediction of slice statistics can be carried out according to the procedure in Section 2.2, and the results of statistics prediction will be used in the denormalization. In denormalization, enhanced SAN denormalizes the output from LSTM by the results of statistics prediction in Section 2.2, and restores non-stationary factors to give an accurate estimation.

Suppose the output sequence ${\left\{{\bar{y}}_{j}\right\}}_{j=1}^{V}$, with forecasting length, as the output from LSTM, where SAN splits the output series into *V* slices that have the same slice length *Q* with normalization. The denormalization procedure can be formulated as

(14) $${\hat{y}}_{j}={\bar{y}}_{j}*({\hat{\sigma }}_{j}+\varepsilon )+{\hat{\mu }}_{j}$$

where ${\hat{\mu }}_{j}$ and ${\hat{\sigma }}_{j}$ are the mean and standard in the output of statistics prediction, respectively, and ${\hat{y}}_{j}$ is the final forecasting output.

In addition, considering that SAN requires the sequence length to be an integer multiple of the slice length, the statistic prediction model cannot handle an indivisible length to obtain an integer amount for complete slices. We can directly calculate the statistic prediction results using the final slice with data points in an incomplete slice. However, the output may show large fluctuation, especially in situations with few data points in the last incomplete slice. Insufficient data points may lead to a non-negligible deviation, which will affect the final forecasting accuracy.

A tail amendment technology is adopted to improve this situation. For a sequence length without an integer multiple, an amendment will be used in the last incomplete slice.

Suppose the last incomplete slice has length *P*, which is less than slice length *Q*; the added technology will allow a modified calculation of the slice as part of the complete length with amendment. The series *X* input as slices will be modified. The modified last slice with amendment includes two parts. The former part is calculated by decaying the estimation of the slice before the last incomplete one. The latter part is calculated by the last incomplete slice with amendment. The data points of two parts after amendment can be formulated as

(15) $$X_{for}^{i}=X_{pre}^{i}*e^{\frac{-(Q-P-i+1)}{Q}},\;P<Q,\;1\leq i\leq Q-P$$

(16) $$X_{lat}^{j}=X_{last}^{j}*e^{\frac{Q-P}{Q}},\;P<Q,\;1\leq j\leq P$$

where $X_{for}^{i}$ is the former part of the modified last slice, and $X_{lat}^{j}$ is the latter part of the modified last slice. The final modified slice consists of the former part and the latter part in a continuous order, as $\left[X_{for}^{i},X_{lat}^{j}\right]$.

Exponential decay in the former part is used to decrease the effects of the data points with long time steps, which indicates that nearer historical data will have a larger effect on the forecasting result. The length of the former part is the same as *Q*-*P*. The length of the latter part is the same as the length of the last incomplete slice. The latter part is calculated with a fixed exponential enlargement. The enlargement is used to emphasize the data in the last incomplete part.

The tail amendment technology can eliminate the unstable effects caused by the last incomplete slice.

The algorithm of the training procedure of ESAN is shown in Algorithm 1.
**Algorithm 1.** Implementation of training procedureSuppose: Input series $X={\left\{x_{j}\right\}}_{j=1}^{R}$; horizon series $Y={\left\{y_{j}\right\}}_{j=1}^{R}$; 1: Calculate slice length *Q* by *L*_1_ filtering 2: Tail amendment technology 3: Initialize parameters ϕ,θ 4: while not converge do 5:   for all input $x_{j}\in X$, horizon $y_{j}\in Y$ do 6:    Compute input statistics $\mu_{j},\sigma_{j}$ by Equation (12) with *Q* 7:    Predict future statistics ${\hat{\mu }}_{j},{\hat{\sigma }}_{j}$ by Equation (3) using $f_{\phi }(*)$
8:    Update ϕ using loss function $l_{sp}$
9:   end for 10: end while             >Training of the statistics prediction model 11: while not converge do 12:   for all input $x^{i}\in X$, horizon $y^{i}\in Y$ do 13:    Compute input statistics $\mu_{j},\sigma_{j}$ by Equation (12) with *Q* 14:    Normalize input series to $\bar{x_{j}}$ by Equation (13) 15:    Forecast ${\bar{y}}_{j}=g_{\theta }({\bar{x}}_{j})$ 16:    Predict future statistics ${\hat{\mu }}_{j},{\hat{\sigma }}_{j}$ by Equation (3) using $f_{\phi }(*)$
17:.    ${\hat{\mu }}_{j}$.detach(), ${\hat{\sigma }}_{j}$.detach()   >Stop-gradient, freeze the statistics prediction model 18:    Denormalize ${\bar{y}}_{j}$ to ${\hat{y}}_{j}$ by Equation (14) 19:    Update θ using loss function $l_{fm}$
20:   end for 21: end while               >Training of the forecasting model

Here, ϕ and θ are weight parameters in the statistic prediction model and forecasting model, respectively.

### 3.3. Forecasting Model

The original forecasting task is split via a two-stage strategy in enhanced SAN. The first stage is intended to learn the general direction and dispersion of future data with the statistic prediction model in enhanced SAN. The second stage aims to perform the sequential prediction of future data with the normalized data from enhanced SAN. LSTM is used for the second stage subtask, with the help of enhanced SAN for an easier subtask, as the LSTM neural network can perform better with stationary data in a forecasting task.

The training procedure includes the statistic prediction model and LSTM forecasting model. Firstly, the features in three typical operating conditions are put into enhanced SAN. Features are normalized by enhanced SAN and the statistic prediction model is trained. Then, normalized data are set as the input of the LSTM forecasting model, and the LSTM model gives the prediction of future data. Next, the prediction from the LSTM model is denormalized by the output of statistic prediction in enhanced SAN. The data after denormalization are the final result of the forecasting model.

After the training procedure, a validation set is used to adjust the learning rate by the error between the prediction and ground truth. Finally, the trained forecasting model generates the forecasting result in the testing set of the dataset.

**Figure 4:**
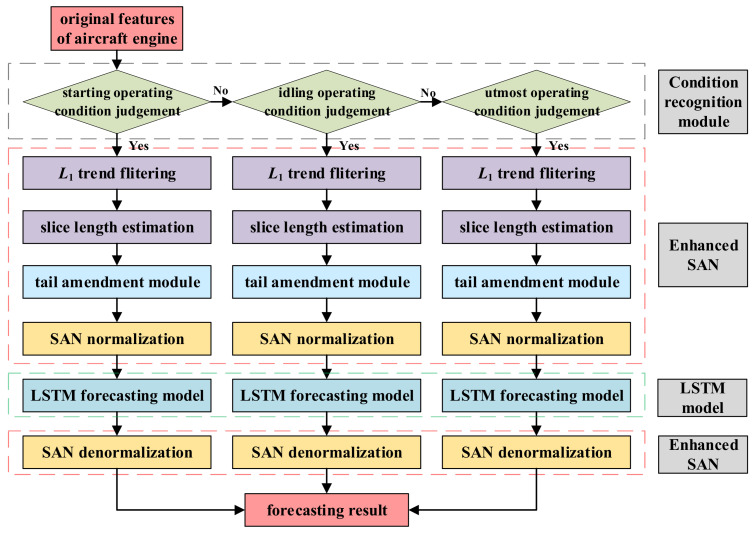
Flowchart of the proposed method.

The flowchart of the proposed method is shown in Figure 4.

## 4. Performance Analysis of Proposed Methods

To validate the proposed method, aircraft engine data are used to analyze the performance of the proposed method.

### 4.1. Aircraft Engine Dataset

**Figure 5:**
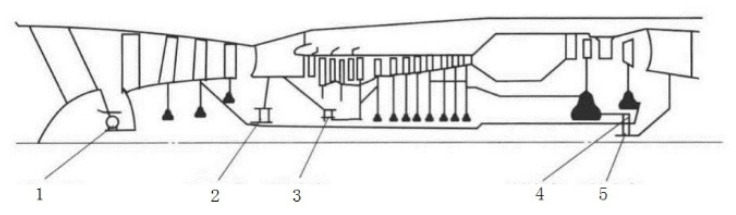
Structure of an aircraft engine.

The dataset comes from the test drive data of a real aircraft engine. The structure of the aircraft engine is shown in Figure 5. In Figure 5, numbers 1–5 indicate the positions of the five main bearings of an aircraft engine.

**Figure 6:**
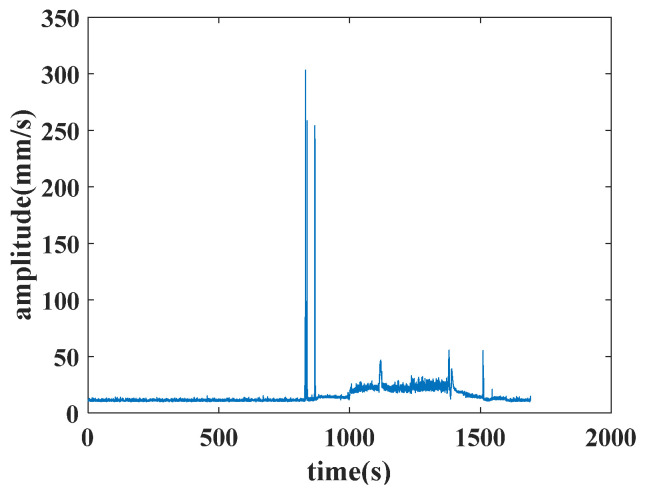
Example of the vibration’s total value in the front axle.

The features of the aircraft engine include 8 features in front axle and 8 features in rear axle, amounting to 16 features. The 8 features in the front or rear axle include the vibration total, vibration component, base harmonic component of high-pressure rotor, second harmonic component of high-pressure rotor, third harmonic component of high-pressure rotor, base harmonic component of low-pressure rotor, second harmonic component of low-pressure rotor and third harmonic component of low-pressure rotor. An example of the vibration’s total value in the front axle is shown in Figure 6.

Each data point of the feature is calculated in 0.2 s. The original features of the aircraft engine contain varying operating conditions. The operating conditions of an aircraft engine vary widely, making it impractical to analyze all of them. Thus, this paper selects three typical operating conditions including the idling operating condition, the start operating condition and the utmost operating condition as the representative operating conditions to process. The discriminations of three operating conditions are set as follows.

The discriminations of the idling operating condition include the low-pressure rotor speed varying within a certain range, the high-pressure rotor speed varying within a certain range, and the fluctuation values of both low-pressure rotor speed and high-pressure rotor speed being less than a threshold.

The discriminations of the starting operating condition include the following: the entry mark requires the state mark of the engine state changing from a certain value to another value; the exit mark requires the state mark of the engine state changing from a certain value to another value; and the start operating condition occurs between the entry mark and the exit mark.

The discriminations of the utmost operating condition include the low-pressure rotor speed and high-pressure rotor speed both varying within a same specific range, the range of the throttle lever angle varying within a certain range, and the fluctuation values of the throttle being less than a threshold. Both the idling operating condition and the utmost operating condition also require 20 continuous seconds.

**Figure 7:**
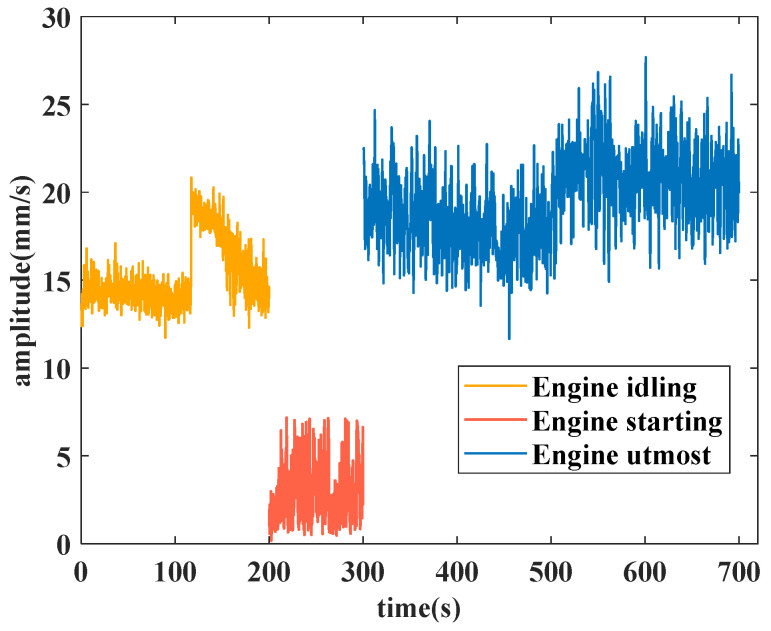
Extracted features in three operating conditions for the experiment.

The analyzed features are the vibration total value in the front axle for the idling operating condition, the second harmonic component value in the high-pressure rotor of the front axle for the start operating condition, and the vibration component value in the front axle for the utmost operating condition. The stitched features extracted from the original features in three typical operating conditions are used for the experiment. The experiment features of the idling operating condition, the start operating condition and the utmost operating condition are depicted in Figure 7.

Each feature in its operating condition has 1000 data points, wherein the idling operating condition calculates a feature data point every 0.2 s, the starting operating condition calculates a feature data point every 0.1 s, and the utmost operating condition calculates a feature datapoint every 0.4 s. Different time intervals are set to ensure that the density of data meet the requirements in model training.

In the training part of the model, three feature data sets are put into the proposed method separately, and we can obtain three models corresponding to these three different operating conditions. In the idling operating condition, the training dataset is from 0 to 166 s. In the starting operating condition, the training dataset is from 0 to 71 s. In the utmost operating condition, the training dataset is from 0 to 314.4 s.

**Figure 8:**
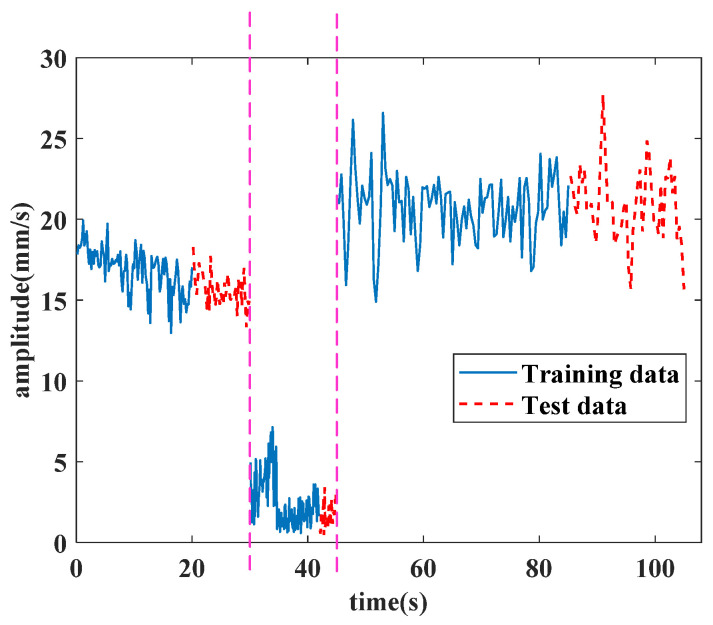
Stitched data with three operating conditions for the forecasting test.

In the test part of the model, a stitched signal combined with three operating conditions is given to verify the validity of the proposed method. A training partial sequence and the test part of the experiment data are shown in Figure 8.

The three operating conditions’ data are separated by a pink dashed line; the first part is the idling operating condition, while the second part is the starting operating condition, and the third part is the utmost operating condition. The partial training part data, positioned at the end of the training part, are set as the test input for the forecasting result. The test data in Figure 8 are the sequences followed by respective training parts in chronological order. The condition recognition technology identifies the operating condition by this discrimination. Firstly, the technology identifies if the starting operating condition is met or not. Then, it calculates the data in 20 continuous seconds to find the idling operating condition and the utmost operating condition. The values of continuous seconds are adjustable. Besides this, the starting operating condition is also judged by its discrimination. The test parts are the real data used for forecasting, and the lengths of the forecasting sequence are 10 s under the idling operating condition, 3 s under the starting operating condition and 20 s under the utmost operating condition. All the experiments were implemented in Python 3.8 and PyTorch 1.9.0, and were conducted on a single NVIDIA RTX 960 8 GB GPU. The operating system was Windows 10.

### 4.2. Validation on Tail Amendment Technology

**Figure 9:**
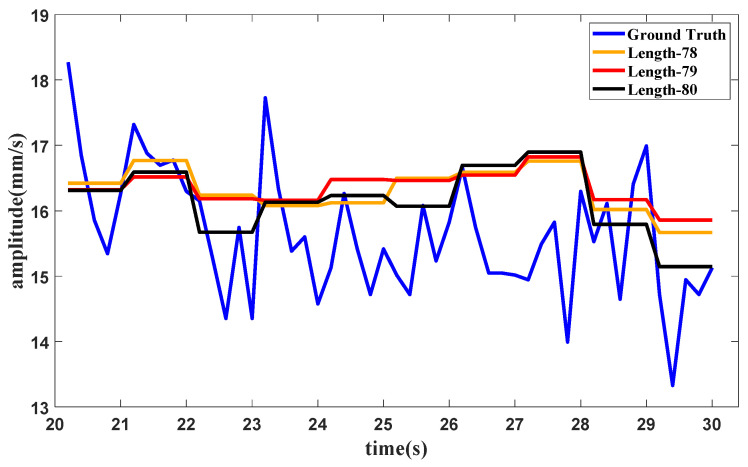
Forecasting results with different sequence lengths.

Furthermore, to validate the behavior of the tail amendment technology in enhanced SAN, an experiment with different sequence lengths is carried out. The dataset comprises the data under the idling operating condition. The same settings are used in this experiment as were used in Section 3, except for sequence lengths, which were set as 78, 79 and 80, respectively. The slice length is 5 and the only sequence length in the integer multiple is 80. Enhanced SAN with sequence lengths of 78 and 79 can also be calculated after amendment. The forecasting results for different length are shown in Figure 9.

It can be seen from the results that datasets with different sequence lengths can achieve approximate performance. The purpose of this amendment technology is to expand the application range of ESAN so that it can handle data with various lengths. The difference between the results is mainly caused by the sequence length itself, which may also be related to the application of the decay part to some extent. In general, the amendment technology can give a robust and accurate prediction, and the application of the amendment technology can extend the use range of enhanced SAN.

### 4.3. Ablation Study

**Figure 10:**
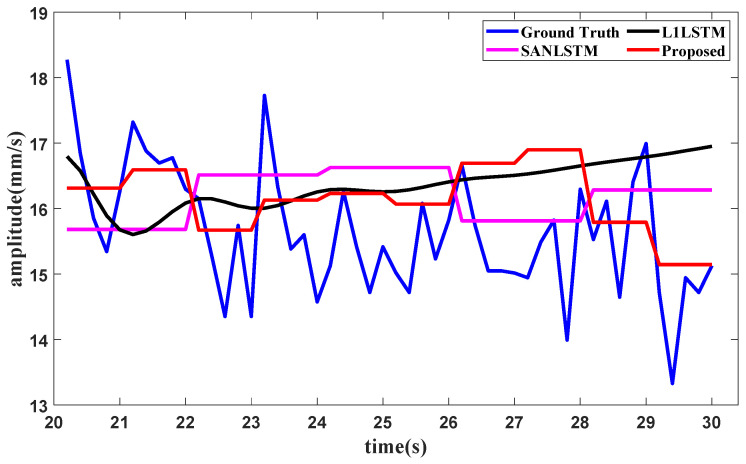
Ablation forecasting result for the idling operating condition.

**Figure 11:**
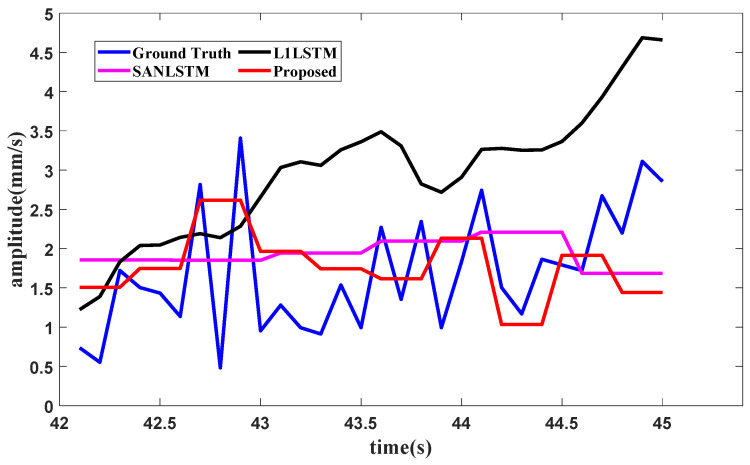
Ablation forecasting result for the starting operating condition.

**Figure 12:**
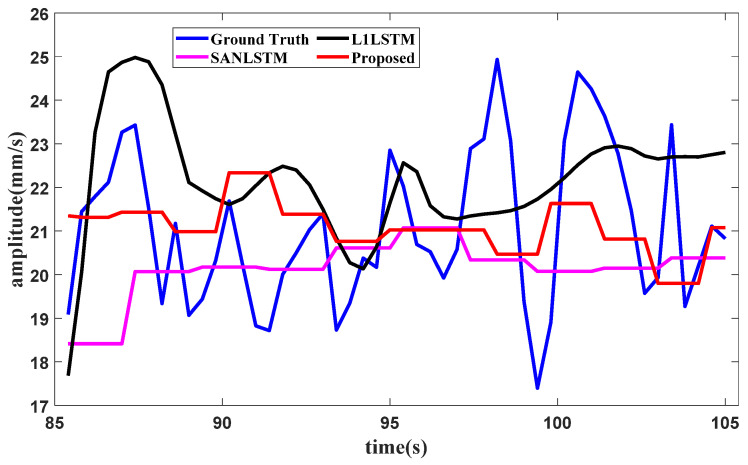
Ablation forecasting result for the utmost operating condition.

To evaluate the effectiveness of the components of the proposed method, an ablation study with the aircraft engine signal dataset shown in Section 3 is conducted. The same experimental settings as those in Section 3 are used in this study. The forecasting results are here generated under three typical operating conditions. The vibration total value in the front axle is chosen for the idling operating condition, the second harmonic component value in the high-pressure rotor of the front axle is chosen for the start operating condition, and the vibration component value in the front axle is chosen for the utmost operating condition. The comparison methods involve conducting ablation studies on the method components for SAN or *L*_1_. The comparison methods used in the ablation study include SAN+LSTM and *L*_1_+LSTM. In SAN+LSTM, the slice length of SAN is set as one of the experimental values, such as 5, 8 or 10. In the proposed method, the slice lengths of enhanced SAN are set to match the amounts of fixed points in *L*_1_ filtering, where the slice lengths in the idling operating condition and the starting operating condition are both set as 5, and the slice length in the utmost operating condition is set as 3. The forecasting results of the proposed method and the comparison methods are shown in Figure 10, Figure 11 and Figure 12, respectively.

Root Mean Square Error (RMSE) and Mean Absolute Percentage Error (MAPE) are used to measure the forecasting error. RMSE is the root of the mean error in the square. MAPE is the average relative error with an absolute value as a percentage. The smaller the RMSE or MAPE, the higher the accuracy of the forecasting result. An error table of RMSE and MAPE for the comparing methods used in the ablation study is shown in Table 1. The least error in each indicator is bold in the table.

The difference between the result and error for SAN+LSTM and the proposed method shows that *L*_1_ filtering benefits the method with SAN, not only via the trend extraction of the filtering, but also via the quantitative estimation of slice length. It indicates that SAN can provide a better prediction that follows the fluctuation of distribution. Therefore, the proposed method can achieve better forecasting results with enhanced SAN.

### 4.4. Comparison of Forecasting Performance of Different Methods

To show the superiority of the proposed method, three other methods have been employed as comparison methods, namely, SAN+DLinear [31], Transformer [33] and Informer [34]. In SAN+DLinear, the slice length of SAN is set with the experimental values 5, 8 and 10, and the result with the least error is chosen for comparison. In the proposed method, the slice lengths of enhanced SAN are set to match the amounts of fixed points in *L*_1_ filtering, where the slice length in the idling operating condition and the starting operating condition are both set as 5, and slice length in the utmost operating condition is set as 3. The batch size is set as 64 in the idling operating condition and the starting operating condition, and in the utmost operating condition, the batch size is set as 32. The learning rate is set as 0.055 and the early stop patience is set as 5.

**Figure 13:**
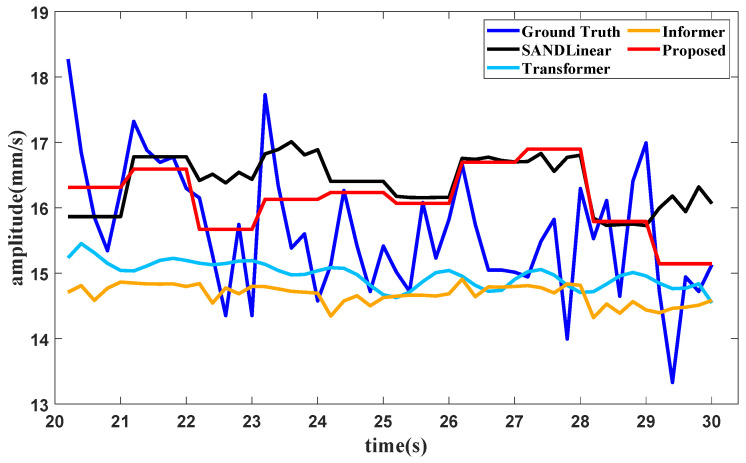
Forecast results of the vibration total value in the front axle produced with different methods.

Suppose that the partial training data in Figure 8 are used to test the forecasting method; when the training data in the first part in Figure 8 are subjected to the proposed method, the condition recognition technology identifies the operating condition as idling, with data provided in 20 s. The forecasting method requires the last 16 s of data for the lookback window, and the forecast result of the total vibration value in the front axle under the idling operating condition is shown in Figure 13.

**Figure 14:**
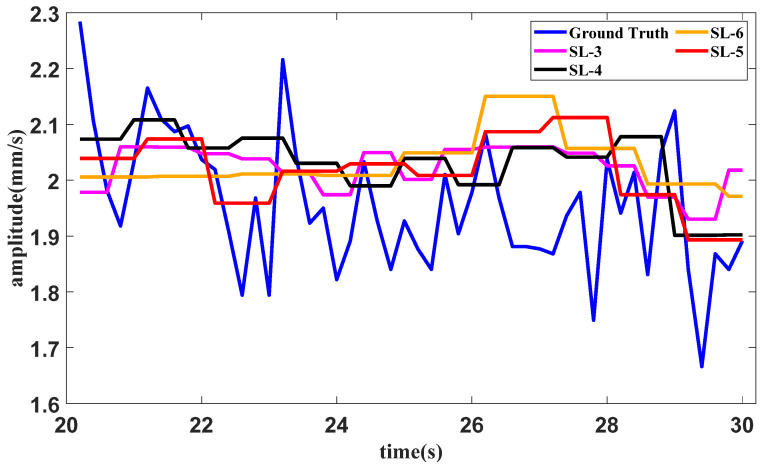
Forecast results of the idling operating condition with different slice lengths.

A test of the method with different slice lengths under the idling operating condition is conducted. In the proposed method, the slice length is set as 5. The slice lengths are set as 3, 4 and 6 for the methods using SAN, LSTM and tail amendment technology. The results of the methods with different slice lengths in the idling operating condition are shown in Figure 14. 

The error table of the method in the idling operating condition with different slice lengths is shown in Table 2. The lowest error for each indicator is bold in the table.

**Figure 15:**
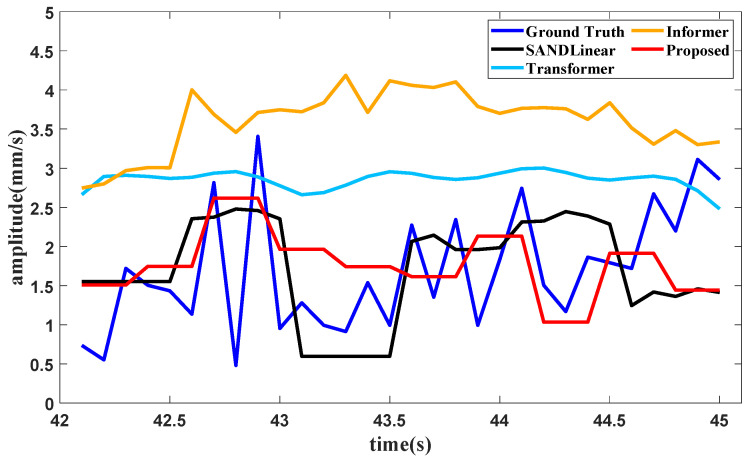
Forecasting result of the second harmonic component in the high pressure rotor in the front axle.

When it comes to the training data of the second part, the condition recognition technology identifies the operating condition as starting from 30 s to 42 s. The forecasting method needs the last 6 s of data for the lookback window, and the forecasting result of the second harmonic component value in the high-pressure rotor of the front axle under the starting operating condition is shown in Figure 15.

**Figure 16:**
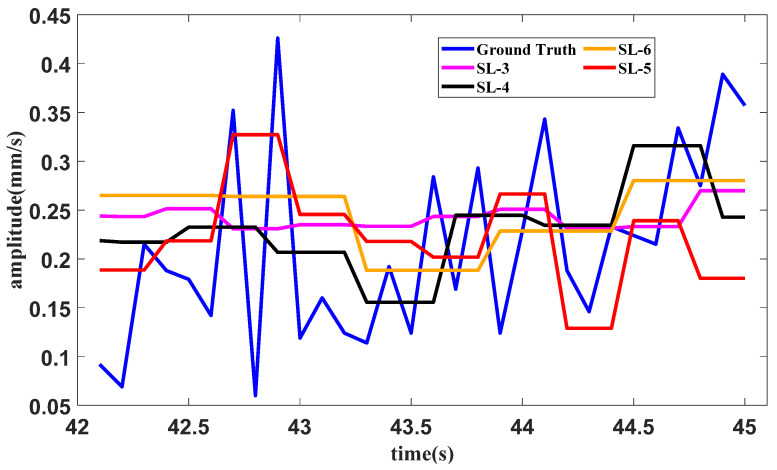
Forecasting result of the starting operating condition with different slice lengths.

Similarly, a test of forecasting with different slice lengths under the starting operating condition is conducted. In the proposed method, the slice length is set as 5. The slice lengths are 3, 4 and 6 in the methods containing SAN, LSTM and tail amendment technology. The forecasting result is shown in Figure 16.

The errors of different slice lengths in the starting operating condition are shown in Table 3. The least error in each indicator is bold in the table.

**Figure 17:**
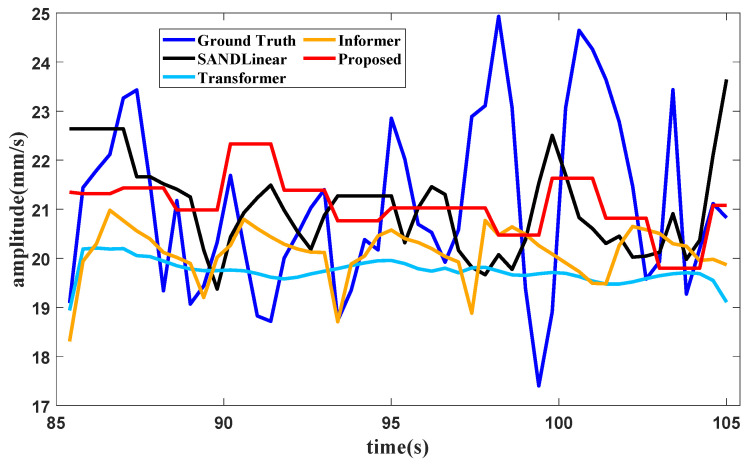
Forecasting result of the vibration component value in the front axle.

When the training data of the third part are input, the condition recognition technology identifies the operating condition as the utmost with data in 20 s, and the data from 45 s to 85 s are all in the utmost operating condition as well. The forecasting method requires the last 32.4 s of data for the lookback window, and the forecasting result of the vibration component value in the front axle under the utmost operating condition is shown in Figure 17.

**Figure 18:**
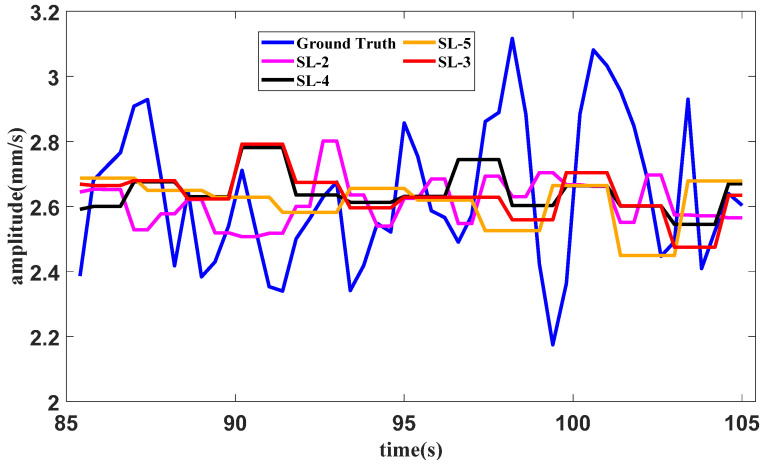
Forecasting result of the utmost operating condition with different slice lengths.

Similarly, a test of forecasting with different slice lengths in the starting operating condition is conducted. In the proposed method, the slice length is set as 3. The slice lengths are further set as 2, 4 and 5 in the methods that contain SAN, LSTM and tail amendment technology. The forecasting result is shown in Figure 18.

The errors associated with different slice lengths in the utmost operating condition are shown in Table 4. The least error in each indicator is bold in the table.

It can be seen from Figure 14, Figure 16 and Figure 18, as well as Table 2, Table 3 and Table 4, that the proposed method with the quantitative estimation of slice length by *L*_1_ filtering produces the lowest error in half of the ratios. The slice length with the lowest error in the starting operating condition is 4, which is close to the estimation of 3 given by *L*_1_ filtering.

The RMSE and MAPE values of different methods of forecasting under three operating condition are shown in Table 5. The least error in each indicator is bold in the table.

It can be seen from Figure 13, Figure 14 and Figure 15 and Table 5 that several forecasting methods can basically forecast the features of the vibration signal under the three typical operating conditions of an aircraft engine. The results of Transformer and Informer remain relatively stable. The reason may be that the requirement for a large amount of data is not satisfied, and the standard deviation in normalization is fairly small, affecting the final result. The resultng figures show that SAN+DLinear and the proposed method can produce better predictions under relative operating conditions. It can also be seen in Table 2 that the proposed method can achieve almost the highest accuracy compared with other forecasting methods under different operating conditions.

## 5. Conclusions

In this study, a trend forecasting method for the vibration signal of an aircraft engine is proposed by combing enhanced SAN using LSTM under multi-operating conditions. The condition recognition technology can identify the idling operating condition, the starting operating condition and the utmost operating condition automatically. To alleviate non-stationary factors in the data, an enhanced SAN is adopted to address the non-stationary data distribution issue, in which *L*_1_ filtering is used to extract the trend part of the signal. Furthermore, *L*_1_ filtering gives a quantitative estimation of slice length in enhanced SAN using the fixed points, given the greater specificity of the current data compared with previous values. A tail amendment technology is applied to expand the application range of the enhanced SAN. The forecasting results under multi-operating conditions are produced by the forecasting model using LSTM with enhanced SAN. A performance analysis of the proposed method with aircraft engine vibrational signals is conducted to verify the validity of the proposed method, and the following conclusions can be obtained:

1. Enhanced SAN with L1 filtering can alleviate non-stationary data effectively, which benefits the forecasting model. The proposed method can forecast the vibrational data in aircraft engines well;
2. The tail amendment technology used in the last incomplete slice can achieve a closer result compared with the complete one, widening SAN’s application range;
3. The proposed method can achieve higher forecasting accuracy compared with other forecasting methods for aircraft engine features, which shows the effectiveness and superiority of the proposed method.

## Figures and Tables

**Table 1:** Error of compared methods for the ablation study.

Condition Method	The Idling		The Starting		The Utmost	
	RMSE	MAPE	RMSE	MAPE	RMSE	MAPE
SAN+LSTM	1.263	6.994	0.858	38.052	2.141	7.650
*L*_1_+LSTM	1.315	7.191	1.504	64.068	2.151	9.030
Proposed	**1.060**	**5.480**	**0.832**	**36.011**	**1.834**	**6.905**

**Table 2:** Errors of methods with different slice lengths in the idling operating condition.

Indicator	Slice Length			
	3	4	6	5
RMSE	0.138	0.136	0.155	**0.133**
MAPE	5.917	5.754	6.780	**5.480**

**Table 3:** Error of methods with different slice lengths in the starting operating condition.

Indicator	Slice Length			
	3	4	6	5
RMSE	0.099	**0.096**	0.103	0.104
MAPE	35.810	**31.823**	37.870	36.011

**Table 4:** Errors of methods with different slice lengths in the utmost operating condition.

Indicator	Slice Length			
	2	4	5	3
RMSE	0.229	0.229	0.241	**0.227**
MAPE	**6.812**	6.905	7.238	7.109

**Table 5:** Errors of the different forecasting methods.

Condition Method	The Idling		The Starting		The Utmost	
	RMSE	MAPE	RMSE	MAPE	RMSE	MAPE
SAN-DLinear	1.269	6.877	0.897	37.978	2.093	7.989
Transformer	1.118	**5.426**	1.402	70.396	2.180	7.578
Informer	1.343	6.746	2.089	100.531	1.994	6.951
Proposed	**1.060**	5.480	**0.832**	**36.011**	**1.834**	**6.905**

## Data Availability

The data cannot be made publicly available upon publication because they contain commercially sensitive information. The data that support the findings of this study are available upon reasonable request from the authors.
